# Supplementary material for: Carboxymethyl-Dextran-Coated Superparamagnetic Iron Oxide Nanoparticles for Drug Delivery: Influence of the Coating Thickness on the Particle Properties
Source: Int J Mol Sci. 2022 Nov 25;23(23):14743. doi: 10.3390/ijms232314743 (PMC9740466; doi:10.3390/ijms232314743)
Supplement: Supplementary file 1 [file ijms-23-14743-s001.zip › ijms-2052762-supplementary.pdf]

Carboxymethyl-dextran coated superparamagnetic iron oxide nanoparticles for drug delivery: Influence of coating thickness on the particle properties

Chiara Turrina[a], Davide Milani[a], Anna Klassen[a], Diana M. Rojas-González[b], Jennifer Cookman[c], Matthias Opel[d], Barbara Sartori[e], Petra Mela[b], Sonja Berensmeier[a] and Sebastian P. Schwaminger[a,f*]

[a] Bioseparation Engineering Group, Department for Engineering and Design, Technical University of Munich

[b] Chair of Medical Materials and Implants, Department of mechanical engineering, Munich Institute of Biomedical Engineering, TUM School of Engineering and Design, Technical University of Munich

[c] Department of Chemical Sciences, Bernal Institute, University of Limerick, Castletroy, University of Limerick, V94 T9PX

[d] Walther-Meißner-Institut, Bayerische Akademie der Wissenschaften, Garching, Germany

[e] Institute of Inorganic Chemistry, Graz University of Technology, Stremayrgasse 9/IV, Graz, 8010, Austria

[f] Division of Medicinal Chemistry, Otto Loewi Research Center, Medical University of Graz, Neue Stiftingtalstraße 6, Graz, 8010, Austria

* sebastian.schwaminger@medunigraz.at

Electronic Supplementary Information

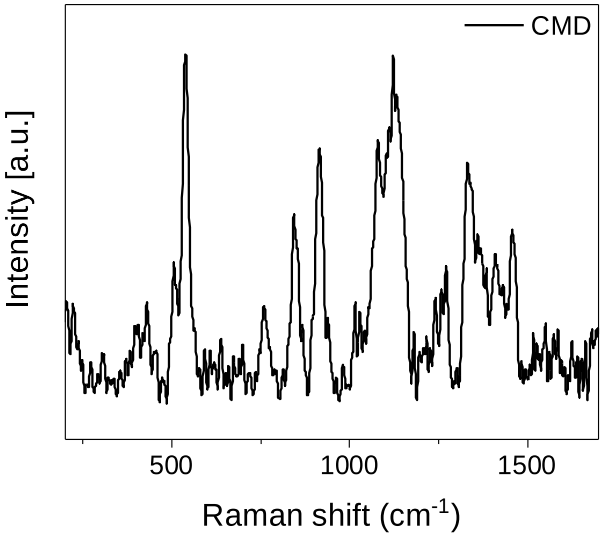

***SI-Figure S1**: **Raman Spectra of CMD** measured using a 488 nm laser, the laser power was reduced to 1 mW for each measurement by optical filters (Exposure: 10 s, Co-Ad. 2).*

Magnetite content was calculated using the formula determined by Schwaminger et al. [1].

$$Magnetite\ (\%) = 100 * (1 - \frac{A(710)}{A(660)}) \quad (1)$$

SI-Table S1: Magnetite contents *of the particles BIONs, ION@CMD6.25 to 125 determined by comparing the magnetite peak area (660) and maghemite peak area. The magnetite content could not be determined for ION@CMD250 because no peak was visible in the Raman spectrum. Peak areas were determined using the Voigt fit at 660 cm^{-1} and 710 cm^{-1}.*

Particles	A(660 cm^{-1})	A(710 cm^{-1})	Magnetite [%]
BIONs	961.8	811.4	15.6
ION@CMD6.26	550.1	434.5	21.0
ION@CMD12.5	805.2	531.8	33.9
ION@CMD25.0	566.6	297.6	47.5
ION@CMD125	418.5	245.2	41.6
ION@CMD250	-	-	-

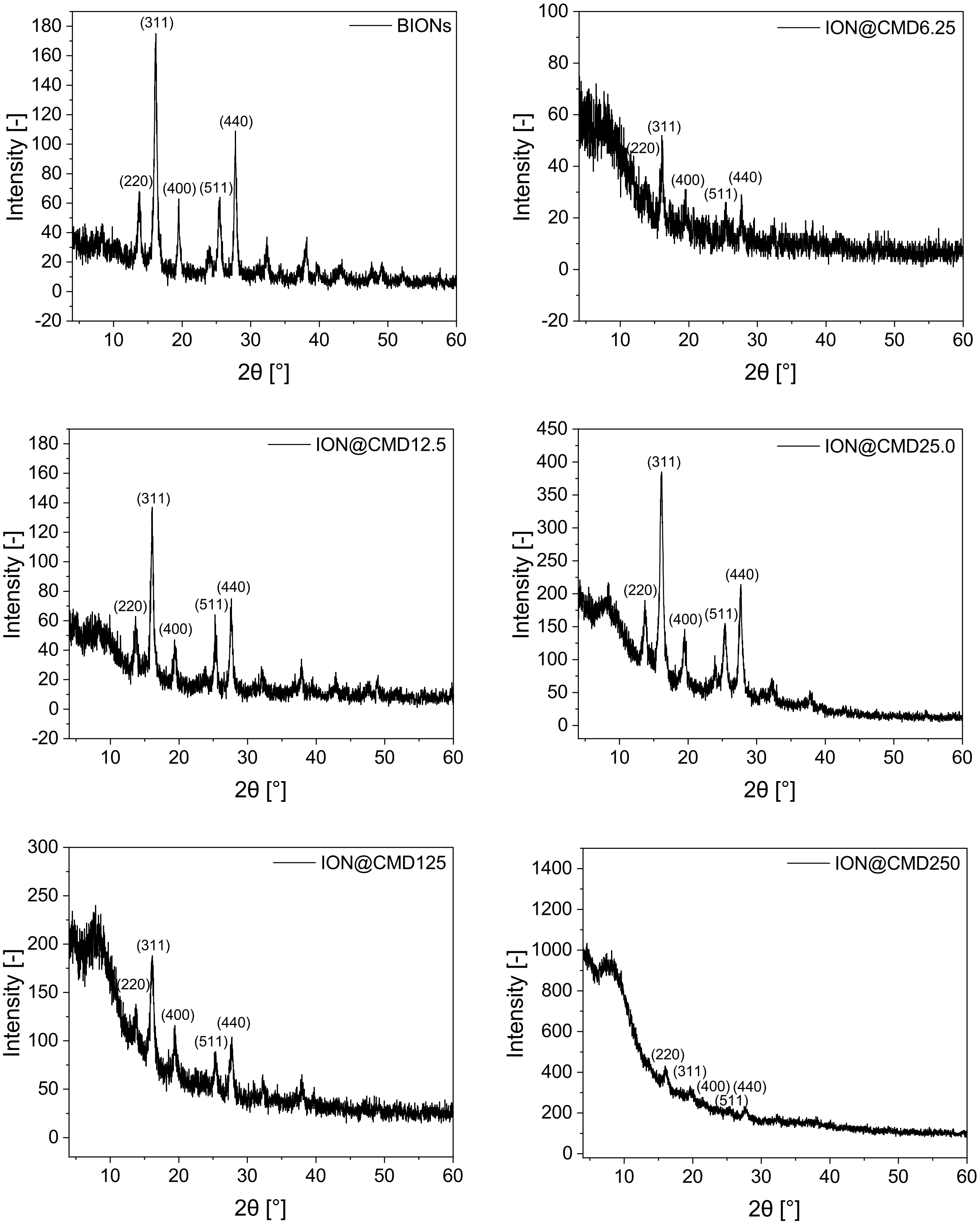

SI-Figure S2: *Diffractograms of BIONs, ION@CMD6.25, ION@CMD12.5, ION@CMD25.0, ION@CMD125, and ION@CMD250. The intensities of the X-ray beam scattered by the sample are plotted as a function of the 2Θ diffraction angle.*

Hydrodynamic diameter d_H was calculated using the Einstein-Stokes-Law for spheres. (Hydrodynamic diameter d_H, diffusion coefficient D, Botzmann constant k_B, temperature T, viscosity η)

$$D = \frac{k_B * T}{6 * \pi * \eta * d_H} \tag{2}$$

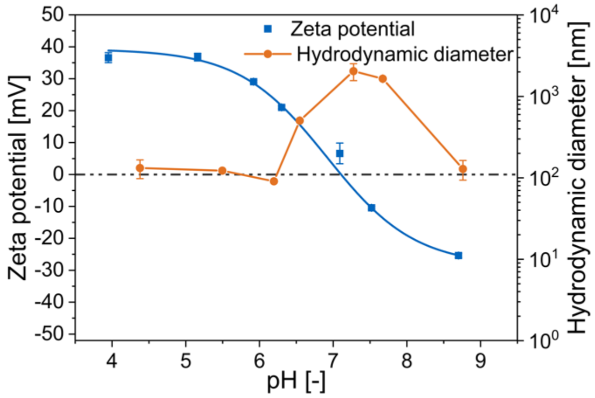

(a)

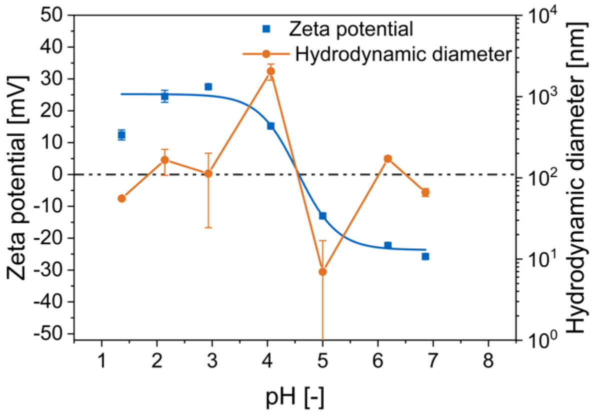

(b)

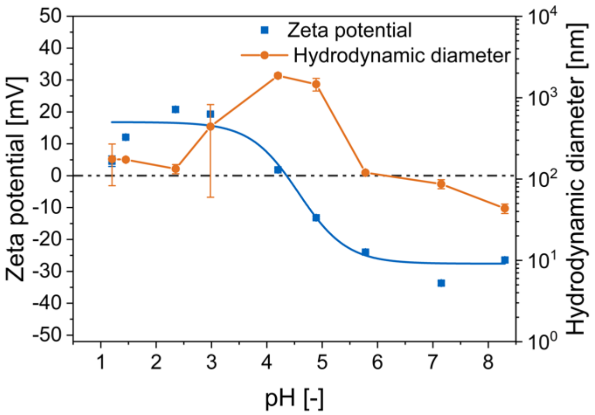

(c)

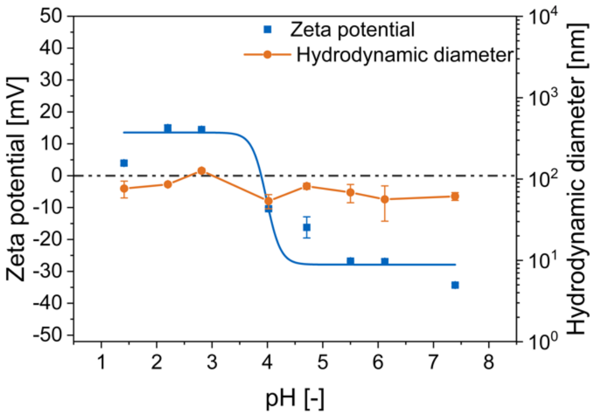

(d)

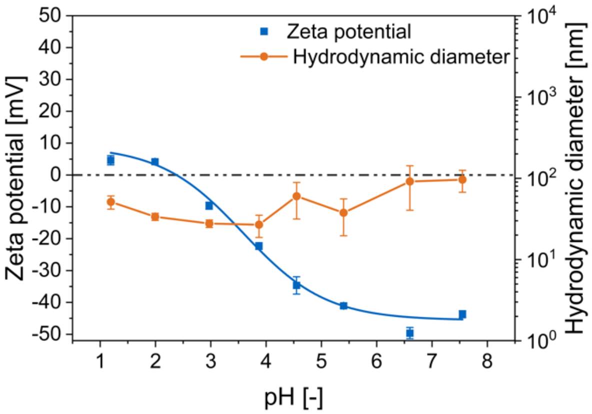

(e)

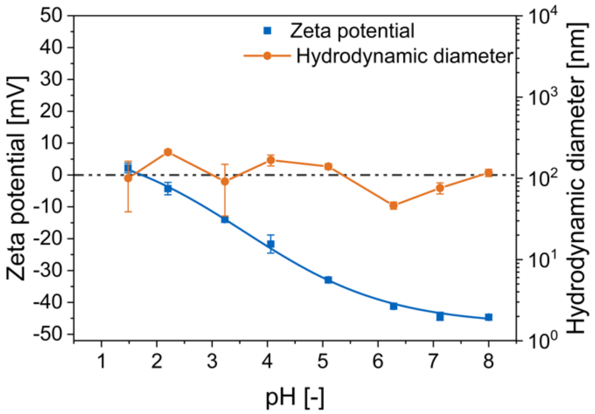

(f)

SI-Figure S3: *Increasing CMD coating thickness shifts the IEP in the acidic. IEPs and d_H through different pH values of a) BIONs, b) ION@CMD6.25, c) ION@CMD12.5, d) ION@CMD25, e) ION@CMD125, and f) ION@CMD250.*

Small Angle X-Ray Scattering data were acquired at the Austrian SAXS beamline at the Elettra Synchrotron in Trieste; the beamline length was set to 1386.101 mm, corresponding to a q range of 0.07 nm^{-1} - 5.3 nm^{-1},where $q = 4\pi \sin\theta/\lambda$, λ is the wavelength of the incident X-rays, and 2θ is the scattering angle. The photon energy was set to 8 keV corresponding to a wavelength of 0.154 nm. The sample was loaded in a quartz capillary with 1.5 mm diameter and exposed to X-Rays. 10 images of 10 s each were collected by a Pilatus 3 1M detector (Dectris Ltd., Baden, Switzerland). The angular scale of the diffraction pattern was calibrated with silver behenate (d-spacing 5.8376 nm). The acquired images were azimuthally integrated by SAXSDog, the automatic data integration pipeline available at the SAXS outstation, normalized on transmission and fluctuation of the primary beam intensity, and background subtracted.

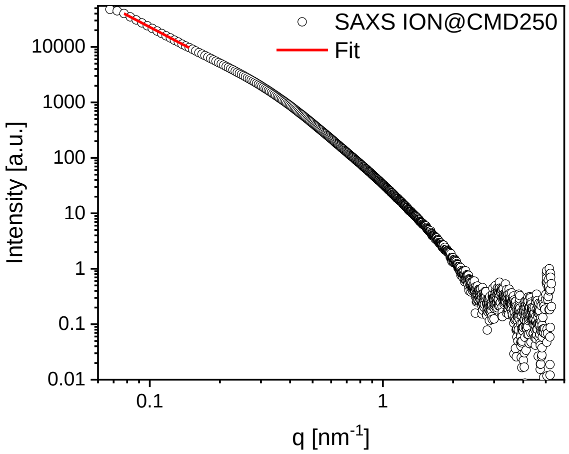

SI-Figure S4: *SAXS profiles of ION@CMD250 and fit at pH 7.*

The Scherrer equation was used to calculate the particle size L of the magnetite crystal. The Scherrer shape factor K has a constant value of 0.89. The X-ray wavelength l is 0.07093 nm. The Bragg angle θ_0 and the full half-width of the reflection $\Delta 2\theta$ are calculated using Origin software. The two largest reflections of the plane (311) and (440) were used

$$D = \frac{K * \lambda}{\beta * \cos(\theta)} \tag{3}$$

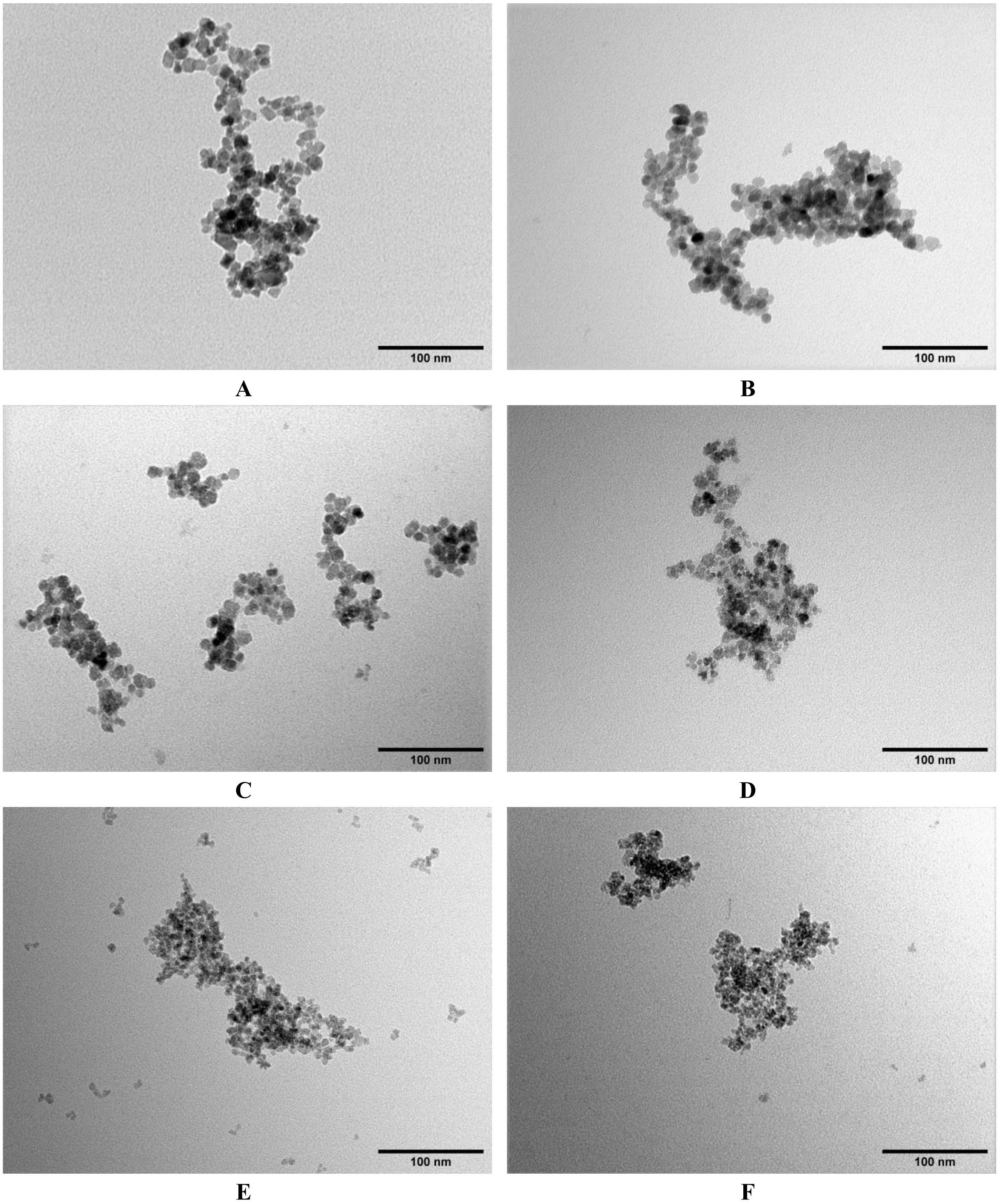

SI-Figure S5: *TEM micrographs at a magnification of 120k of (A) BIONs, (B) ION@CMD6.25, (C) ION@CMD12.5, (D) ION@CMD25, (E) ION@CMD125 and (F) ION@CMD250. Images were processed with ImageJ.*

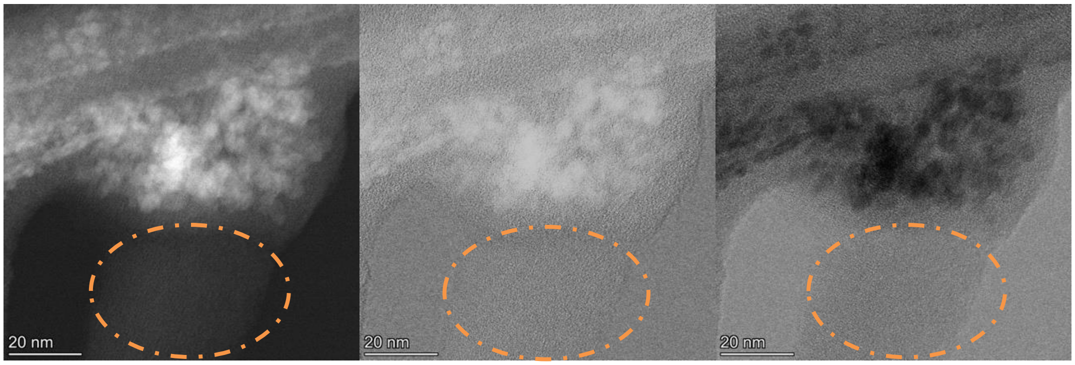

SI-Figure S6: *HAADF-STEM micrographs indicating where the nanoparticle core with an amorphous coating for ION@CMD12.5 on the left. The picture in the middel shows DPCx (A-C) and the right one DPCy (B-D). The orange makers show artifacts created by the rastering electron beam. Scale bars shown inset.*

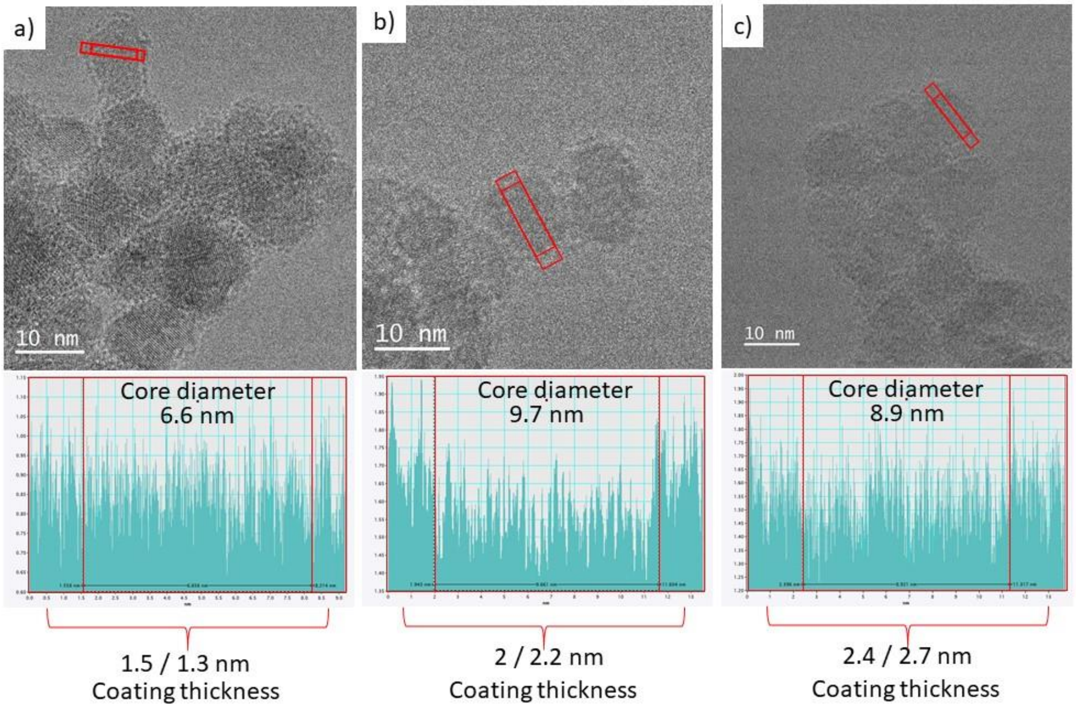

SI-Figure S7: *Low-dose TEM micrographs analyzed for core size and coating thickness for A) ION@CMD250, B) ION@CMD12.5, and C) ION@CMD6.25.*

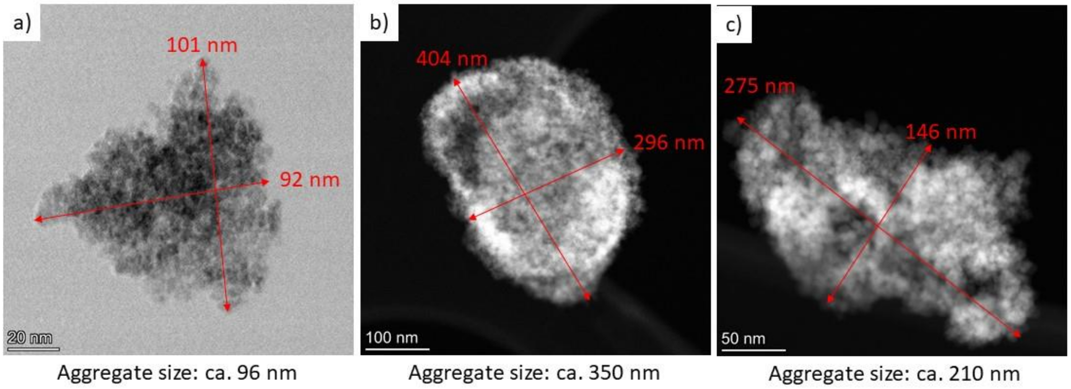

SI-Figure S8: *Low-dose TEM micrographs analyzed for their agglomerate size for A) ION@CMD250, B) ION@CMD12.5, and C) ION@CMD6.25.*

SI-Table S2: *Mean hydrodynamic diameters of BIONs and IONs@CMD in water, 50 mM PBS and human Plasma. For d_H in water, the amount of particles per agglomerate is calculated based on d_{TEM}. For PBS and Human plasma, the stabilization is calculated by dividing through the agglomerates in water.*

**Particles**	**d_H in dH2O [nm]**	**Amount of particles (d_{TEM})**	**d_H in 50 mM PBS (pH 7.4) [nm]**	**$d_{H,PBS}/d_{H,H2O}$**	**d_H in Human Plasma [nm]**	**$d_{H,HP}/d_{H,H2O}$**
BIONs	503 ± 10.5	57.3x	1902 ± 360	3.78x	442 ± 9.63	0.87x
ION@CMD6.26	137 ± 0.33	12.6x	165 ± 12.83	1.21x	50.6 ± 2.46	0.37x
ION@CMD12.5	87.1 ± 10.9	7.99x	158 ± 5.47	1.81x	41.5 ± 9.47	0.48x
ION@CMD25.0	94.2 ± 8.07	11.8x	73.6 ± 9.59	0.78x	79.3 ± 39.0	0.84x
ION@CMD125	162 ± 101	21.5x	34.5 ± 0.81	0.21x	51.6 ± 15.6	0.32x
ION@CMD250	200 ± 32.3	31.6x	69.3 ± 12.3	0.35x	25.8 ± 3.34	0.13x

LangevinMod Fit

$$y = y_0 + C\left(coth\left(\frac{x - x_C}{s}\right) - \frac{s}{x - x_C}\right)$$

$$cothz = \frac{e^z + e^{-z}}{e^z - e^{-z}}$$

SI-Equation 4:*Modified Langevin function by OriginLab, with y_0 = offset, x_c = center, C = Amplitude, s = Scale. Lower and upper bounds: none.*

SI-Table S3**:** *Sedimentation velocity of BIONs and ION@CMDs in dH2O (pH = 7 – 7.4). Measurements were taken at wavelengths of 870 nm, 630 nm, and 420 nm (Profile: 1000; Interval: 1s; Angle: 0°; Light factor: 1.00; Temperature: 25 °C).*

Particles	Sedimentation velocity [µm s^{-1}]
BIONs	1152
ION@CMD6.25	78.95
ION@CMD12.5	18.39
ION@CMD25.0	30.72
ION@CMD125	90.24
ION@CMD250	160.6

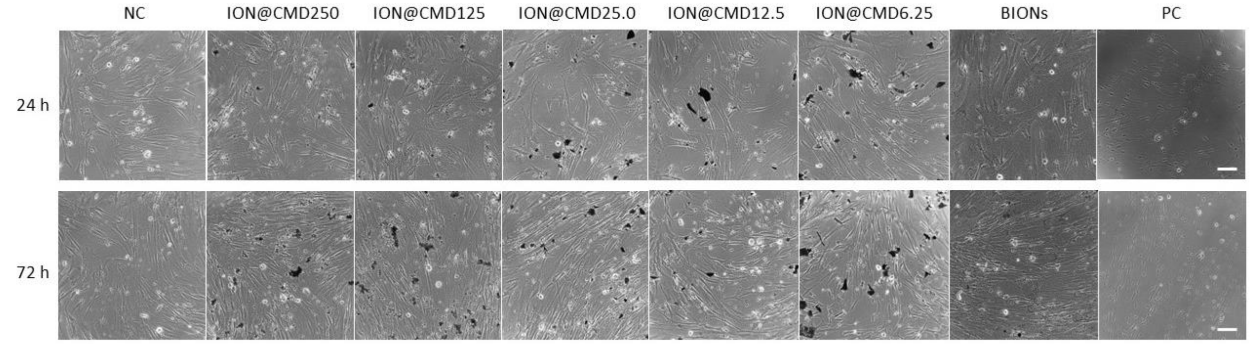

SI-Figure S9: *Phase contrast images of the cells incubated with IONs@CMD and BIONs after 24 and 72 hours for the cytocompatibility assay. Scale bar: 100 µm.*

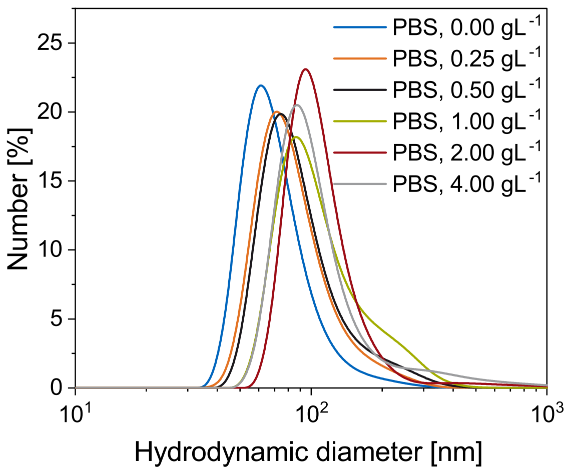

(a)

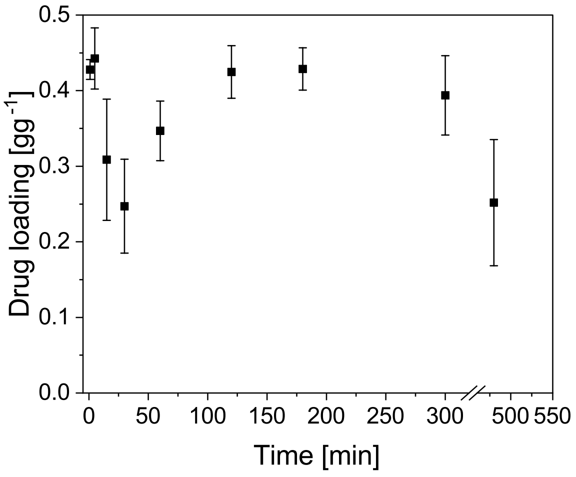

(b)

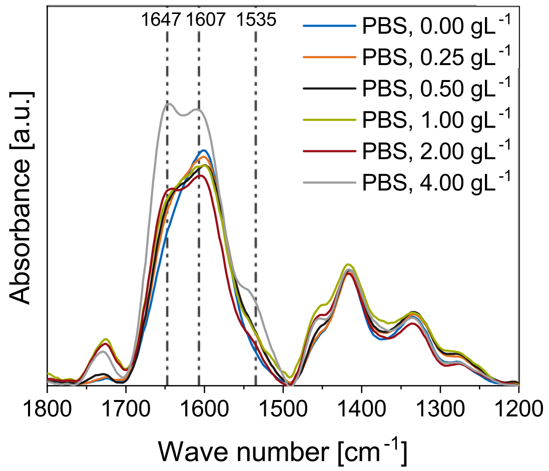

(c)

SI-Figure S10: *Adsorption of LL onto the surface of ION@CMD12.5 in 50 mM PBS buffer at pH 7.4.): a) Hydrodynamic diameters of particles after LL adsorption at pH 7.4 in 50 mM PBS buffer. b) Adsorption kinetics of 4.00 g L-1 LL onto the surface of ION@CMD12.5 (1 gL-1) were measured over 8 hours. c) FT-IR spectrum of adsorbed LL (0.00 g L-1 to 4.00 g L-1 used) on ION@CMD12.5 (24 scans).*

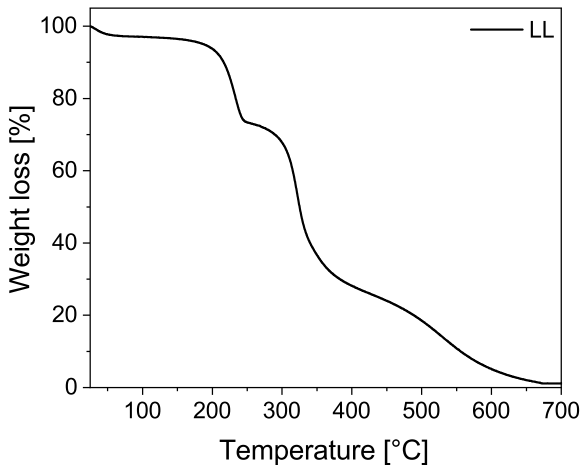

SI-Figure S2: *Weight loss (%) of LL II plotted against Temperature (°C).*

SI-Table S4: *Efficiency of the protocols used to bind LL to the particle surface of ION@CMD12.5. Values were calculated by dividing the amount of LL bound by the amount of LL used. The calculation for Adsorptions are referred to the loadings reached with adsorption. Washing steps were not included.*

**LL used in adsorption [g L^{-1}]**	**Efficiency [%]**	**LL used in covalent binding [g L^{-1}]**	**Efficiency [%]**
0.00	0.00	0.00	0.00
0.25	28.4 ± 1.09	0.80	82.2 ± 0.11
0.50	25.9 ± 1.07	1.00	77.6 ± 0.18
1.00	13.5 ± 3.86	1.50	74.9 ± 0.18
2.00	12.4 ± 0.93	2.00	79.4 ± 0.08
4.00	8.07 ± 1.43	2.50	79.2 ± 0.19

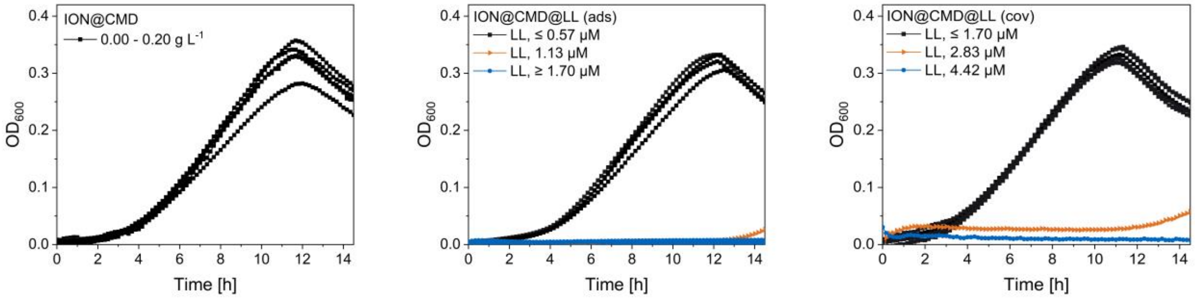

(a) **(b)** **(c)**

SI-Figure S3: *OD600 measurement of E.coli growth of different a) ION@CMD12.5 concentrations and different amounts of ION@CMD@LL obtained by b) adsorption or c) covalent binding.*

After a short lag phase of about two and a half hours, the exponential phase starts about ten hours after the beginning of the experiment. At an OD600 of 0.35, *E. coli* reaches its stationary phase, followed by the death phase. Both measurements at OD600 and the cell counting showed no or negligible influence on cell growth of the ION@CMD12.5.

ION@CMD (0 g/L) ION@CMD (0.01 g/L) ION@CMD (0.03 g/L)

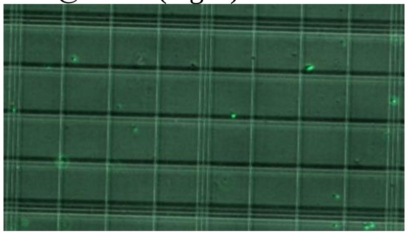
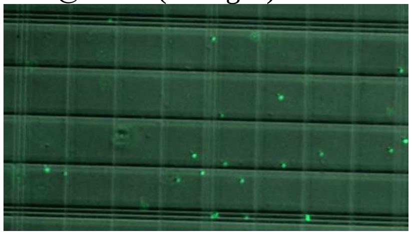
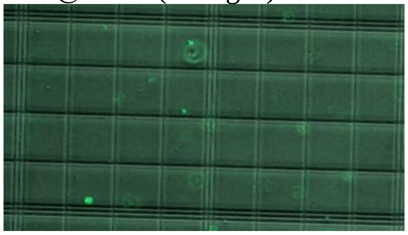

ION@CMD (0.05 g/L) ION@CMD (0.075 g/L) ION@CMD (0.1 g/L)

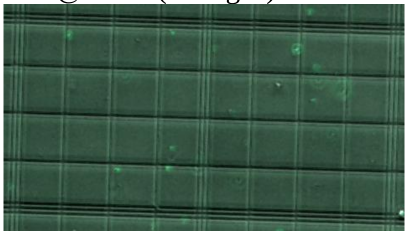
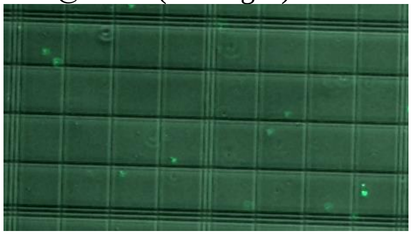
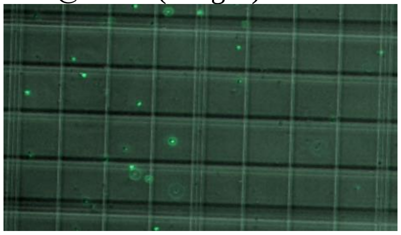

ION@CMD (0.2 g/L) ION@CMD (0.3 g/L) ION@CMD (0.4 g/L)

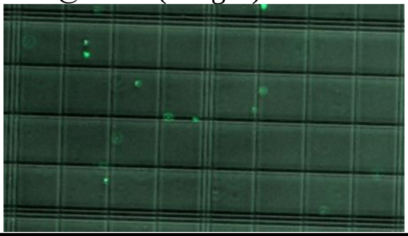
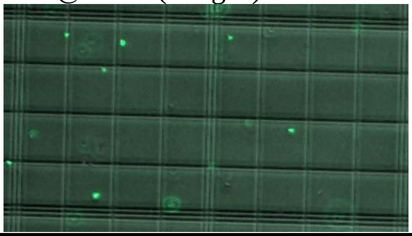
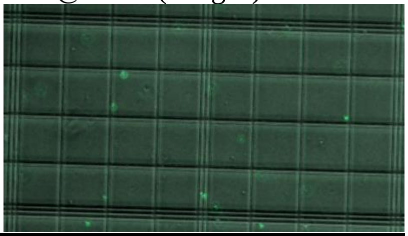

ION@CMD@LL cov (0 µM) ION@CMD@LL cov (0.11 µM) ION@CMD@LL cov (0.28 µM)

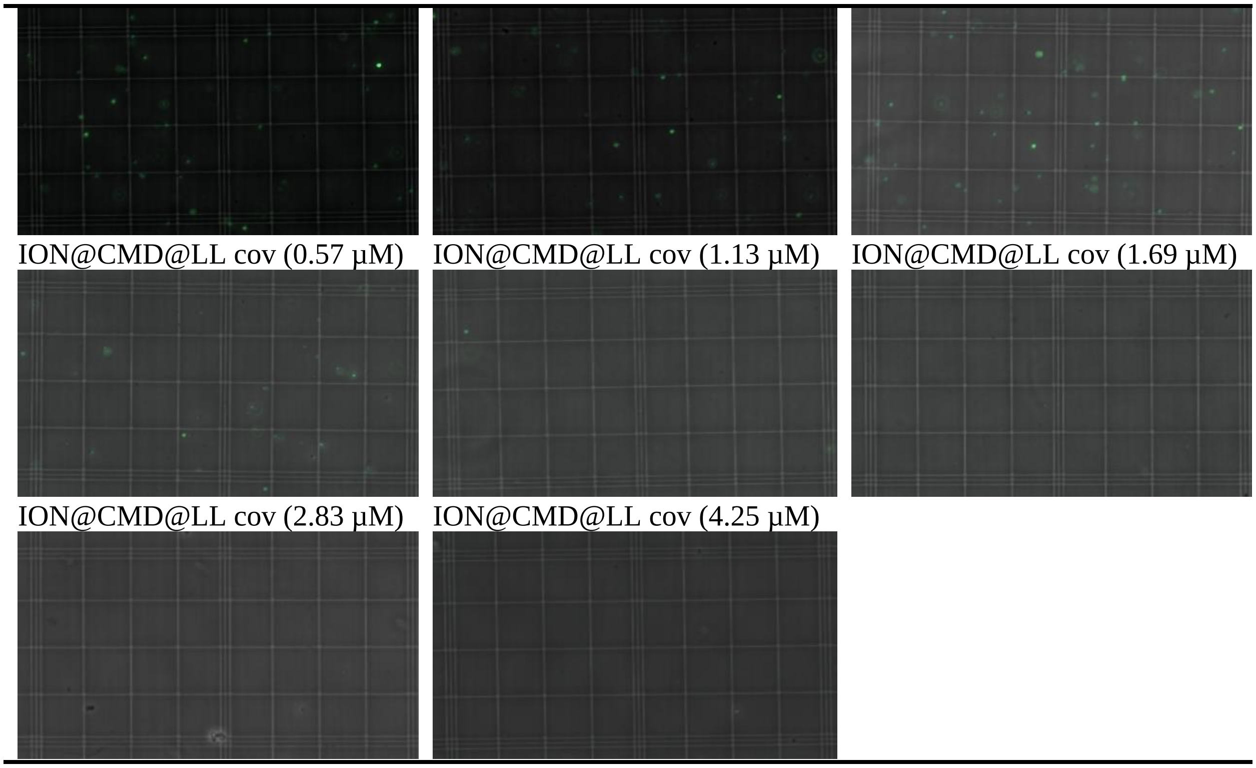

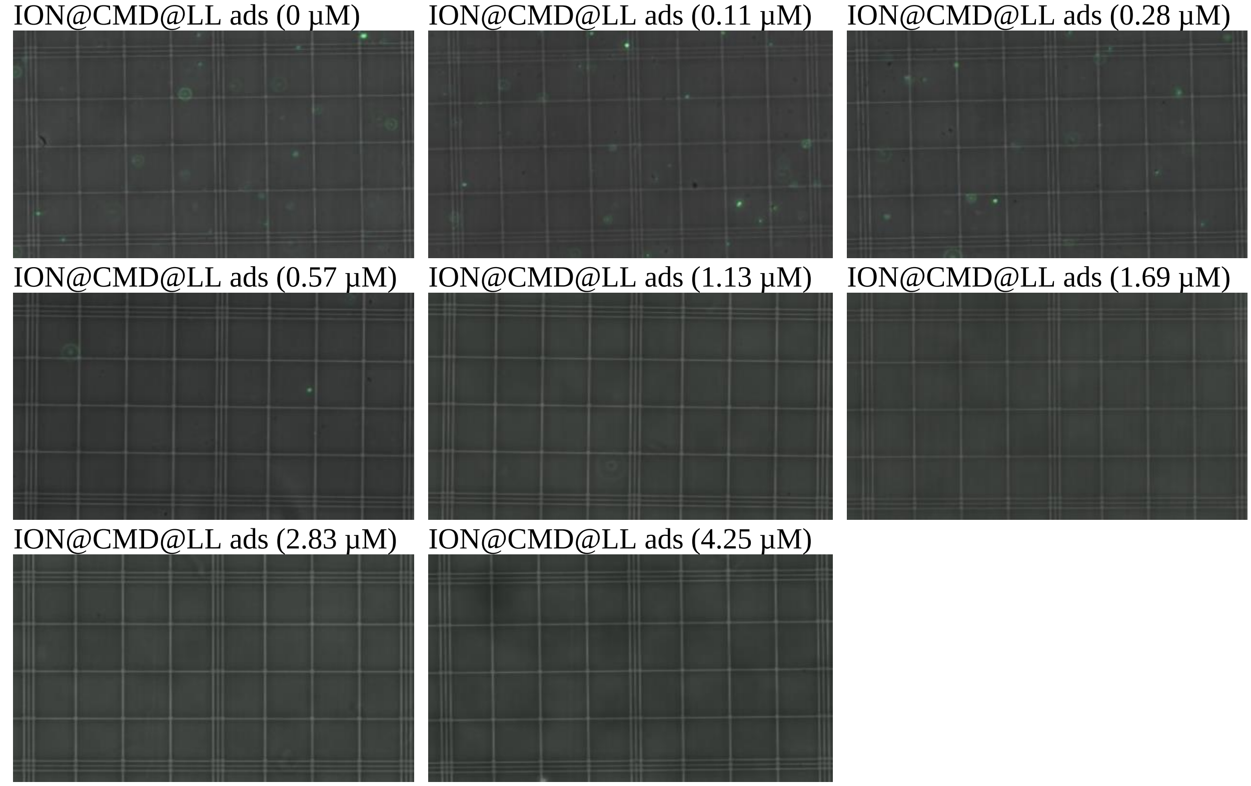

SI-Figure S4*: E.coli colony grown in M9 media with ampicillin, incubated with different ION@CMD12.5, ION@CMD@LL (cov), and ION@CMD@LL (ads) concentrations (37 °C, addition of IPTG after 5 h).*

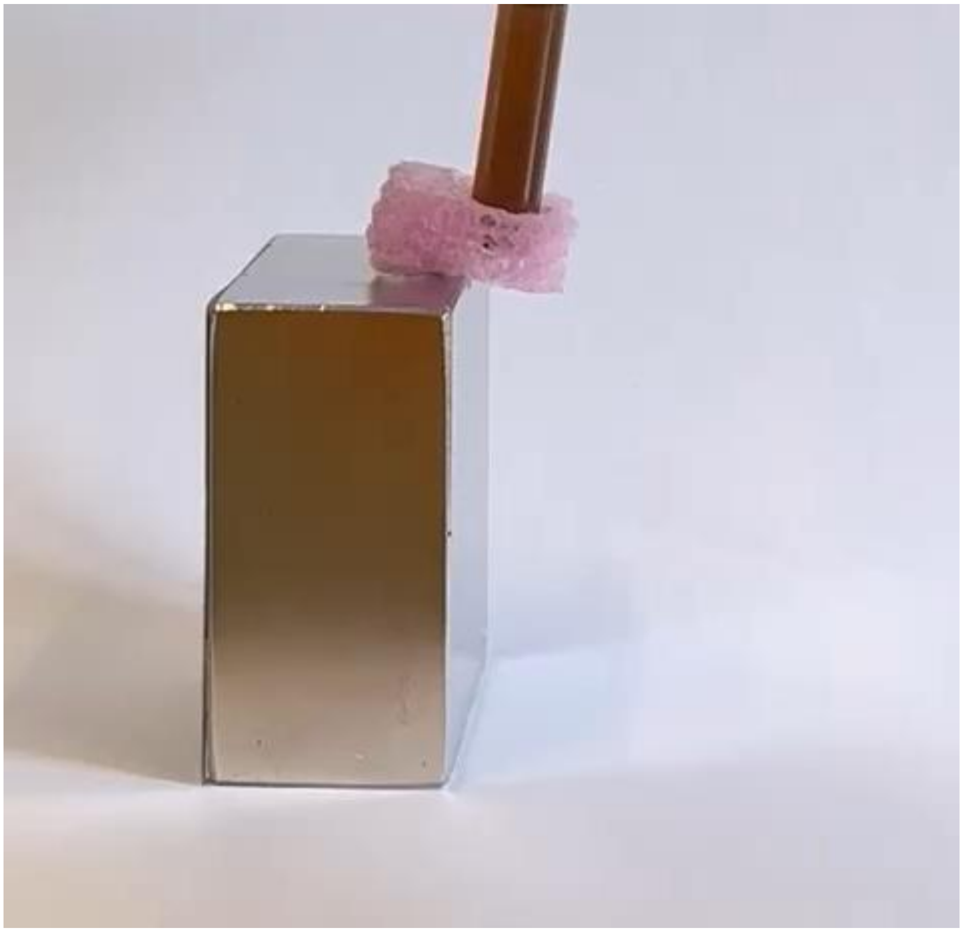

SI-Movie 1: *A$_{1g}$ L$_{-1}$ solution of ION@CMD12.5 with 0.55 g g^{-1} LL contacted to a rectangular magnet (50.8 x 50.8 x 25.4 mm, adhesion force 100 kg, placement force 20 kg, obtained from Supermagnete).*

References

[1] S. Schwaminger, C. Syhr, S. Berensmeier, Controlled Synthesis of Magnetic Iron Oxide Nanoparticles: Magnetite or Maghemite?, Crystals 10 (2020) 214. https://doi.org/10.3390/cryst10030214.
